# Heptannulated Perylene Diimides: Formation and Reactivity of Electron‐Deficient Tropylium Cations and Heptafulvenes

**DOI:** 10.1002/anie.202419899

**Published:** 2024-11-22

**Authors:** Agata Wiencierz, Tadeusz Lis, Piotr J. Chmielewski, Joanna Cybińska, Marcin Stępień

**Affiliations:** ^1^ Wydział Chemii Uniwersytet Wrocławski, ul. F. Joliot-Curie 14, 50–383 Wrocław Poland; ^2^ Polski Ośrodek Rozwoju Technologii (PORT), ul. Stabłowicka 147, 54–066 Wrocław Poland

**Keywords:** perylene diimides, tropylium cations, aromatic substitution, chromophores

## Abstract

The development of new π‐conjugated motifs opens pathways to previously unexplored classes of organic semiconductors and functional dyes. In this study, five‐ and seven‐membered carbocycles were fused at the ortho and bay regions of electron‐deficient perylenes, starting from a common dialdehyde precursor. Structural analysis of the resulting perylene tetraesters, dianhydrides, and diimides (PDIs) revealed three distinct ring‐fusion patterns and defined stereochemistry. The fused PDI cycloheptatrienes demonstrated susceptibility to acid‐catalyzed transarylation, involving tropylium cation intermediates, which can be used preparatively. Under superacidic conditions, the PDI tropylium cations were directly observed and shown to undergo hydride‐transfer reductions. Additionally, a fused PDI bis(heptafulvene) was synthesized by dehydrogenating a suitably substituted PDI cycloheptatriene. The final system contains two quinomethane units, which can be protonated to yield a stable tropylium‐like dication.

## Graphical abstract

Reported herein are three ring fusion patterns from one chemical reaction. Carbocyclic heptannulation provides access to brightly fluorescent, chiral perylene diimides, which can be converted into highly electrophilic tropylium cations and functionalized by direct aromatic substitution reactions.

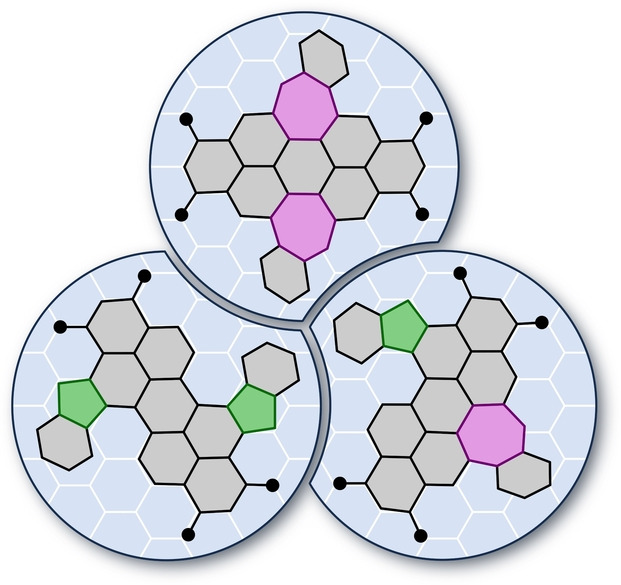

## Introduction

**Figure 1:**
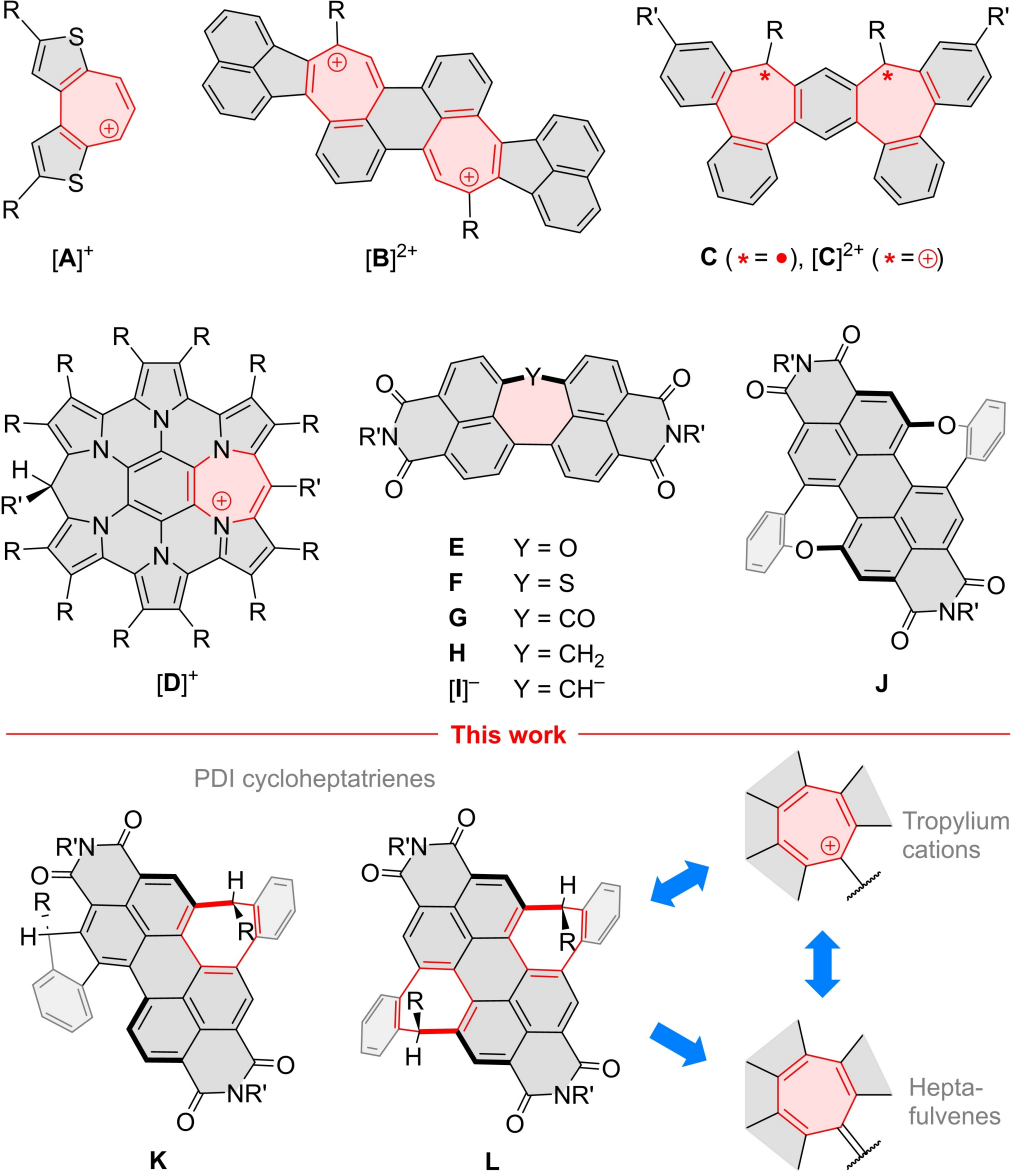
**A**–**D**: examples of frameworks containing tropylium and heterotropylium substructures.[^7^, ^11^, ^13^, ^24^] **E**–**J**: PDI analogues containing 7‐membered rings.[^33^, ^34^, ^35^] **K**–**L**: Bay‐fused PDI cycloheptatrienes as precursors of tropylium cations and tropyl radicals.

Since its first characterization by Doering and Knox,[^1^, ^2^, ^3^] the tropylium cation has remained of continuing interest not only as a non‐benzenoid aromatic motif *par excellence* ^[4]^ but also as a component of more complex π‐conjugated cations, including red and near‐infrared chromophores,[^5^, ^6^, ^7^] porphyrin analogs,[^8^, ^9^] as well as carbocyclic[^10^, ^11^, ^12^] and heterocyclic[^13^, ^14^] nanographenoids (Figure 1). Tropylium derivatives have been useful as organocatalysts,[^15^, ^16^] stimuli‐responsive dyes,^[17]^ clusteroluminogens,^[18]^ G‐quadruplex sensors,^[19]^ and dye‐sensitized solar cells.^[20]^ The tropylium ring and its heterologues may be used not only to impart permanent positive charge to a π system, but also to stabilize curvature and chirality, and act as a precursor to π‐extended tropyl radicals.[^21^, ^22^, ^23^, ^24^, ^25^, ^26^, ^27^, ^28^] Exploration of these possibilities remains challenging, however, because synthetic access to *peri*‐fused 7‐membered rings is often limited by internal strain, susceptibility to rearrangement,^[29]^ and unfavorable reactivity patterns.

Here we report first examples of tropylium cations and heptafulvenes *peri*‐fused to bay regions of perylene diimides (PDIs). PDIs and their analogues have been one of the most‐studied classes of organic dyes,[^30^, ^31^] however, PDI‐based structures containing fused 7‐membered rings remain scarce.[^32^, ^33^, ^34^, ^35^, ^36^, ^37^] The family of ring‐expanded systems developed by Shinokubo et al. (**E**–**H**, Figure 1)[^33^, ^34^, ^35^] is of particular interest, as it contains a fully carbocyclic structure, **H**, which may be viewed as a cycloheptatriene analogue. The latter ring, however, does not produce a tropylium‐like cation but is easily deprotonated to give the corresponding anion, [**I**]^−^, which dimerizes upon oxidation. Seven‐membered rings fused to bay areas are so far limited to azepines^[32]^ and weakly conjugated benzoxepines (**J**)^[36]^ and 1,3,2‐dioxasilepines.^[37]^ In the present work, we develop the fully carbocyclic bay‐fused PDI cycloheptatrienes **K** and **L** and demonstrate their transformations into tropylium cations and redox‐active heptafulvenes.

## Results and Discussion

**Scheme 1:**
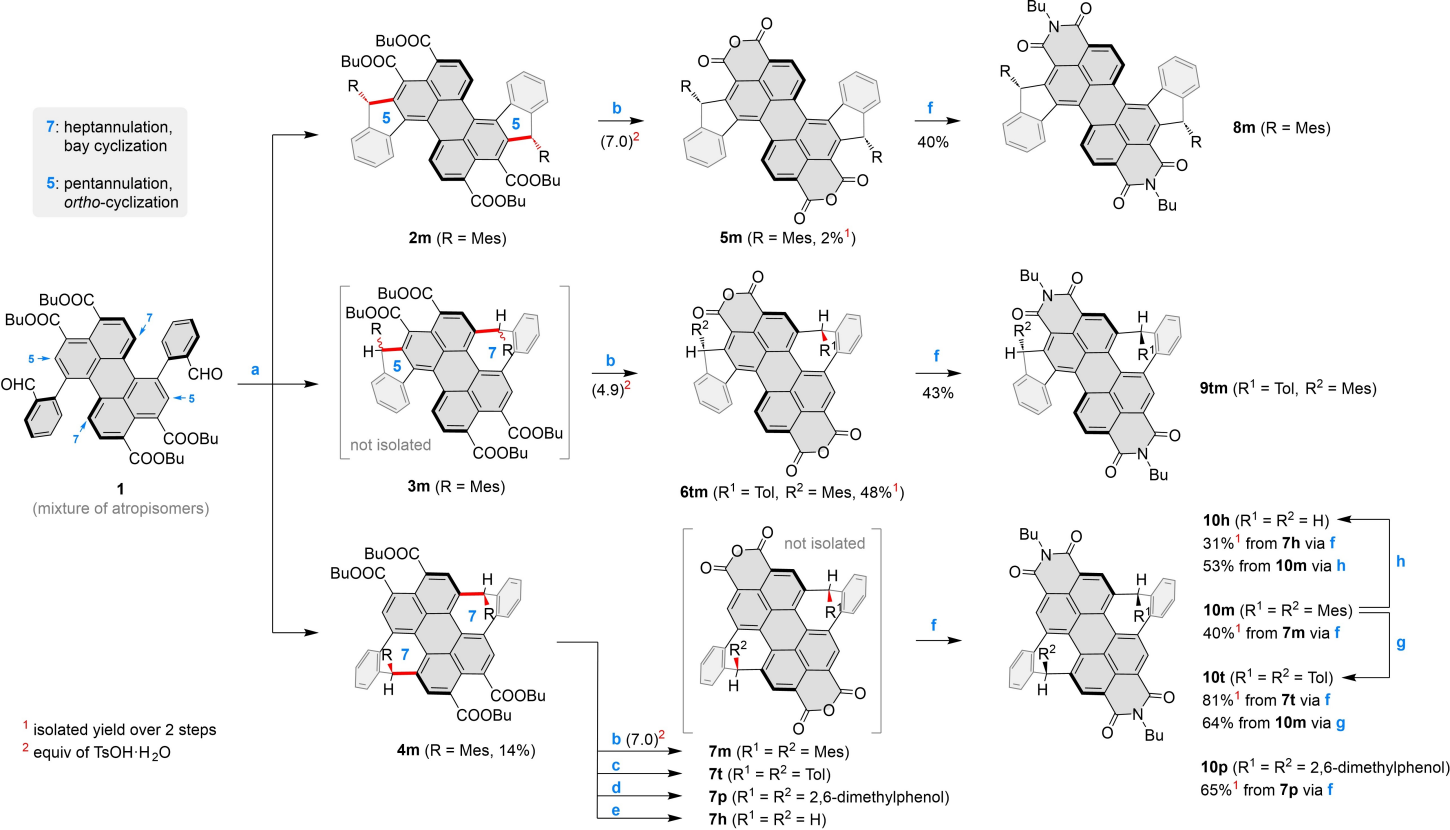
Synthesis of penta‐ and heptannulated PDIs **8–10**. Newly formed C−C bonds are highlighted in red. Reagents and conditions: a) 1. MesMgBr, THF, rt, overnight, 2. BF_3_⋅Et_2_O, DCM, rt, 5 min; b) *p*‐TsOH⋅H_2_O, toluene, 110 °C, 40 h; c) TfOH, toluene, 110 °C, 18 h; d) *p*‐TsOH⋅H_2_O, 2,6‐dimethylphenol, 160 °C, 5 days; e) TfOH, mesitylene, 110 °C, 18 h; f) *n*‐butylamine, *n*‐butanol:water (1 : 1, v:v), 80 °C, 18 h; g) TfOH, toluene (dry), rt, 18 h; h) TfOH (neat), rt, 2 min. Yields of the imidization reaction (step f) were affected by a follow‐up imide–lactam ring contraction^[40]^ (see the Supporting Information).

**Synthesis**. Our work toward heptannulated PDIs started with the dialdehyde tetraester **1** (Scheme 1), itself conveniently made from the corresponding 1,7‐dibromoperylene derivative.^[38]^ As a synthetic precursor, **1** created a possible selectivity challenge, as it could potentially lead to both penta‐ and heptannulated products via *ortho*‐ and bay cyclization, respectively. Indeed, when **1** was subjected to the Grignard addition–cyclization sequence, using MesMgBr as the aryl source (step **a**, Scheme 1), it yielded a mixture of products, from which the desired bis‐heptannulated **4** **m** could be isolated in 14 % yield. A small amount of crude bis‐pentannulated **2** **m** was also obtained, which was transformed into the corresponding dianhydride (**5** **m**) and diimide (**8** **m**). The latter species is structurally reminiscent of a recently reported diindeno‐fused diazacoronene diimide.^[39]^ The inseparable remainder of the cyclization mixture was suspected to contain tetraester **3m**, featuring two different ring sizes, and was treated with *p*‐toluenesulfonic acid monohydrate (TsOH⋅H_2_O) in toluene, with the goal of obtaining the corresponding anhydride. Our hope that the latter derivative would be easier to isolate was indeed fulfilled; however, to our amazement, the resulting anhydride, **6** **tm**, contained two different aryl substituents: a mesityl at the five‐membered ring, and an unexpected *p*‐tolyl group at the seven‐membered ring. **6** **tm** was subsequently converted into the respective diimide, **9** **tm**, featuring the same mixed substitution pattern.

We thought that the substituent replacement observed in **6** **tm** and **9** **tm** had occurred via an acid‐catalyzed transarylation involving the toluene solvent. Further experiments were then performed on **4** **m** to check whether its seven‐membered rings are also susceptible to substitution. Remarkably, **4** **m** underwent only partial transarylation in the presence of TsOH⋅H_2_O, and the reaction could be completely suppressed by increasing the amount of acid, leading to the Mes‐substituted dianhydride **7** **m** (Scheme 1). This counterintuitive behavior was attributed to the increasing amount of water introduced with the acid catalyst. In fact, nearly complete transarylation of **4** **m** could be achieved when anhydrous triflic acid (TfOH) was used instead of TsOH⋅H_2_O, yielding the Tol‐substituted **7** **t**. This tandem transarylation–anhydridization works with other electron‐rich arenes, as exemplified by the reaction of **4** **m** with 2,6‐dimethylphenol, which produced the corresponding anhydride **7** **p**. An interesting exception was however found in a reaction performed with TfOH in mesitylene: instead of the expected **7** **m**, we observed a product of double reductive dearylation **7** **h**. In the above reactions, crude anhydrides were difficult to isolate and had to be directly converted into the corresponding N‐butyl imides, **10** **m**, **10** **t**, **10** **p**, and **10** **h**, which were completely purified and characterized. Importantly, the reactivity seen at the anhydride stage could be reproduced for the imides: specifically, **10** **m** was susceptible to both transarylation and dearylation, yielding respectively **10** **t** and **10** **h**.

**Figure 2:**
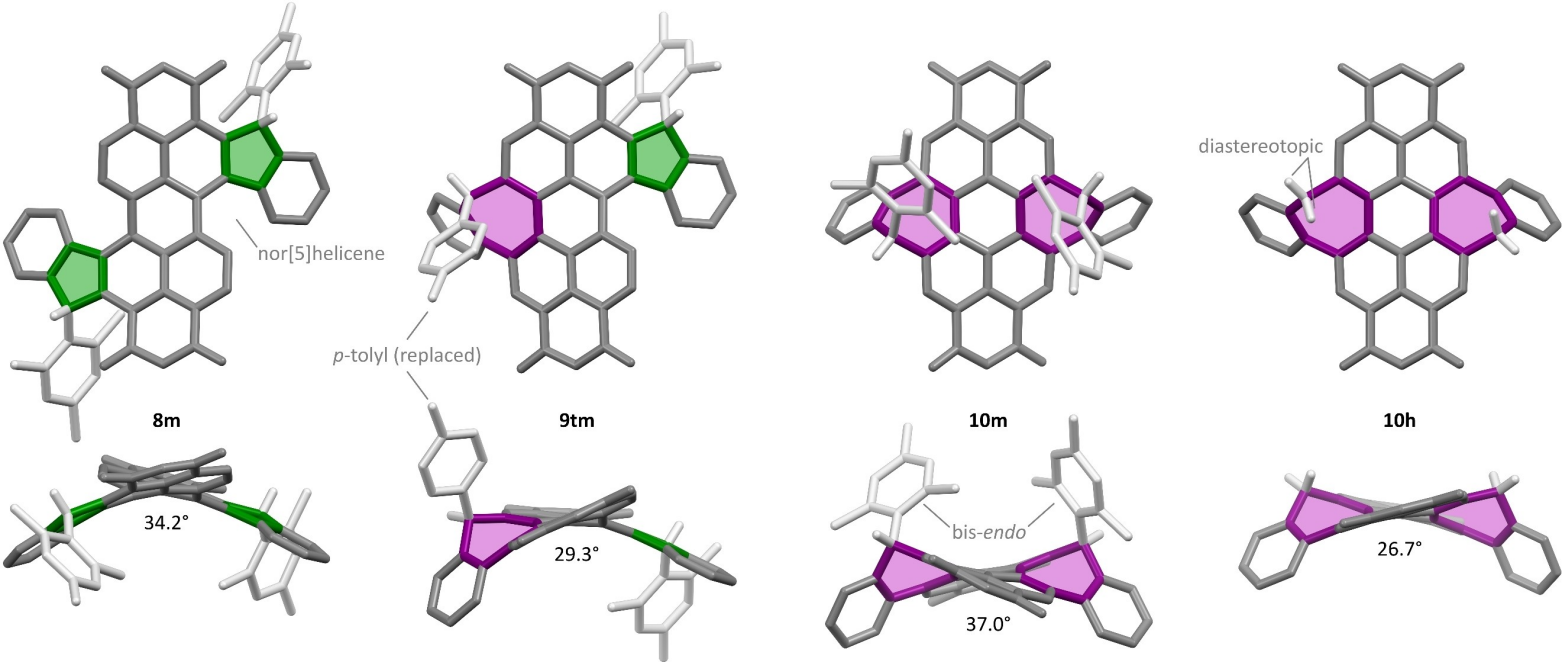
Molecular structures of penta‐ and heptannulated PDIs determined using single‐crystal X‐ray diffraction analyses. Solvent molecules, N‐butyl substituents, and C(sp^2^)‐bound hydrogens are omitted for clarity. Aromatic backbones are shown in gray, while the C(sp^3^) bridges and the attached substituents are shown in light gray. For each structure, the dihedral angle is given between the mean planes of the C_12_NO_2_ naphthalimide subunits.

**Structure and Properties**. The three distinct annulation patterns of PDI derivatives **8–10** and aryl replacement in **9** **tm** were unequivocally confirmed using X‐ray diffraction analyses^[41]^ (Figure 2) and NMR spectroscopy (Supporting Information). Except for **10** **h**, each of these diimides contains two stereocenters, and each was isolated as a racemate of a single chiral diastereomer. The relative configuration of chiral centers in the doubly annulated PDIs is established during the acid‐catalyzed cyclization step and may be further affected by transarylation. Only one diastereomer was observed in the crude **2m** by ^1^H NMR spectroscopy, suggesting that double pentannulation is mostly stereoselective, and the configuration of the tetraester is carried over to the diimide product **8m**. Analogous conclusions can be drawn for the bis‐heptannulated **10m**, which also demonstrates a preference for homochiral stereocenter configurations. These configurations were retained in **10t** and **10p** (see below), implying that transarylation is also stereoselective. A single diastereomer was observed for **6tm** and **9tm** as well. However, since we were not able to purify **3m** sufficiently, it is not known whether the final stereochemistry was already selected upon cyclization.

The bis‐pentannulated **8** **m** had a *C*_2_‐symmetric structure in the solid state, displaying homochiral configurations not only in the C(sp^3^) stereocenters but also in the helicities of the two nor[5]helicene^[42]^ moieties. These features and the steric congestion of peripheral rings lead to a notable twist at the perylene core (34.2°), measured as the dihedral angle between the naphthalimide halves of the PDI. A similar, though somewhat smaller twist (29.3°) was found in the mixed system **9** **tm**, in which the two aryl groups are located on the opposite sides of the π surface. Compound **10** **m** was characterized in two different crystalline polymorphs, both of which contained a *C*_2_‐symmetric configuration, with the aryl groups in the endo positions. The preference for an endo conformation was also observed in the phenol‐containing derivative **10** **p** (see below). **10** **m** is notable for a particularly large twist (37.0°), which is due in part to the double heptannulation and, in part, to the presence of the bulky aryl groups. In comparison, the aryl‐free **10** **h** has the lowest twist (26.7°); however, it remains conformationally rigid, as evidenced by the diastereotopicity of the C(sp^3^)H_2_ bridges seen in its ^1^H NMR spectrum, and the lack of chemical exchange observed in a room‐temperature ROESY spectrum.

**Figure 3:**
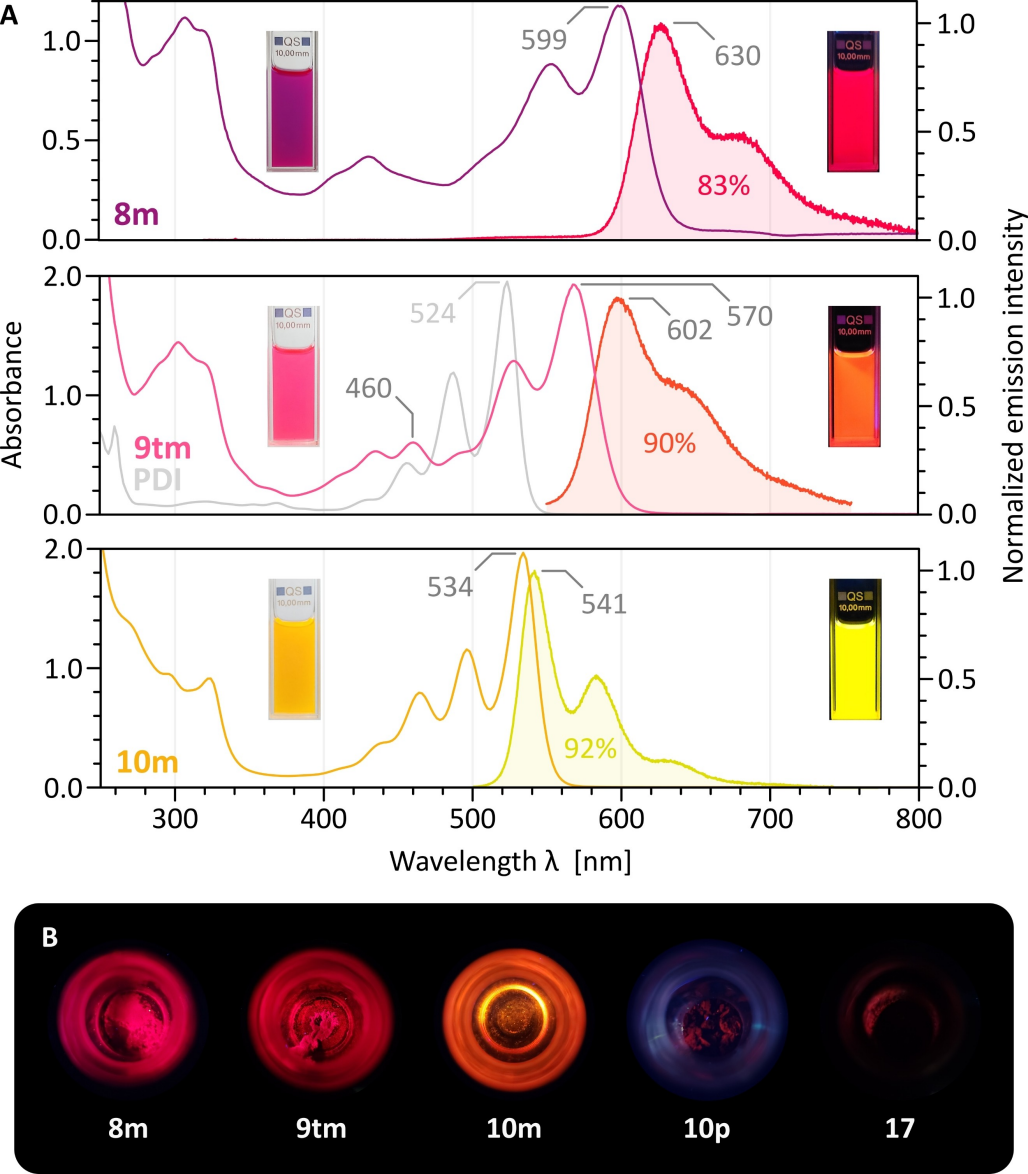
(A) Absorption and fluorescence emission spectra obtained for 8 m, 9tm and 10 m in dichloromethane solutions. The absorption spectrum of N,N′‐di‐(1‐ethylpropyl)‐PDI (gray trace) is shown as a reference. Positions of absorption and emission maxima are indicated with gray labels. Quantum yields are shown as colored labels on the corresponding emission profiles. (B) Fluorescence emission observed for solid powders under UV irradiation.

Compounds **8–10** are brightly colored and strongly fluorescent, yielding PDI‐like absorption and emission spectra (Figures 3A and S112–S118). The S_0_→S_1_ transitions are similar to those of fusion‐free PDIs but are observably red‐shifted. Interestingly, a much larger red shift is caused by ortho‐pentannulation, with the largest *λ*_max_^abs^=599 nm recorded for **8** **m**. Steady‐state fluorescence spectra recorded for the three diimides showed that the pentannulated systems **8** **m** and **9** **tm** produced broader emission bands and larger Stokes shifts than the bis‐heptannulated **10** **m**. These differences imply a higher rigidity of the latter diimide, likely achieved by ring fusion in both bay regions. In all cases, the fluorescence quantum yields are in the range of 80–90 %, with lifetimes in the 6.5–7.4 ns range (Supporting Information). Compounds **8m**, **9tm**, and **10m** are also emissive in the solid state, with an apparent slight red‐shift of the fluorescence color (Figure 3B). In contrast, **10p** is only faintly fluorescent, apparently indicating a charge transfer process involving the phenol substituents. Chiroptical properties of the annulated diimides will be the subject of a subsequent report.

**Scheme 2:**
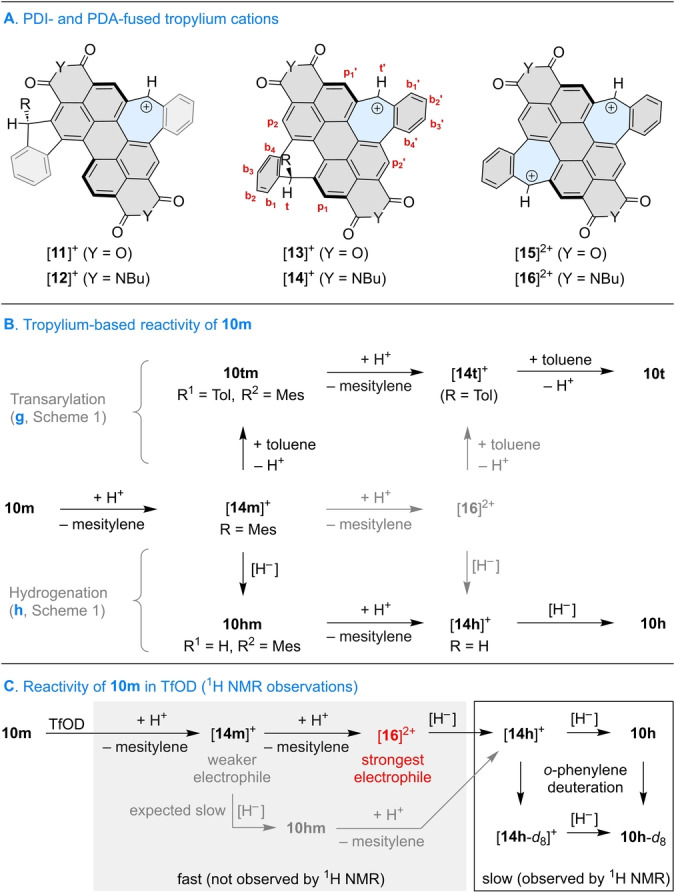
Perylene dianhydride‐ and perylene diimide‐fused tropylium cations and their reactivity. Reversibility of steps and additional protonation of intermediates are not indicated. [H^−^] indicates a formal hydride source.

**Tropylium cations as transarylation intermediates**. The serendipitous discovery of aryl replacement in heptannulated PDIs is preparatively useful, because it can be used for late‐stage functionalization, as shown by the example of the phenol‐bearing **7** **p** and **10** **p** derivatives. The process is however also interesting from the mechanistic viewpoint. The lack of replacement at five‐membered rings, e.g. in **5** **m** or **6** **tm**, suggests that the reaction may proceed with the intermediacy of tropylium cations ([**11**]^+^ – [**14**]^+^, Scheme 2A) and perhaps even dications ([**15**]^2+^, [**16**]^2+^). Because of their local 6‐electron aromaticity, these tropylium species are expected to form more readily than the isomeric fluorenyl cations derivable from, e.g., **5** **m** and **8** **m**, thus determining the selectivity of aryl replacement.

The possible role of tropylium cations is illustrated for the case of **10** **m** in Scheme 2B. Under Brønsted‐acid catalysis, *ipso*‐protonation of the mesityl substituent should lead to mesitylene cleavage and the initial formation of the [**14** **m**]^+^ cation. The latter species may revert to **10** **m** or take one of three forward paths: (a) reaction with another arene,[^17^, ^43^, ^44^] (b) further ionization to yield the bis‐tropylium dication [**16**]^2+^, and (c) reaction with a formal hydride source to produce the singly dearylated intermediate **10** **hm** (R^1^=H, R^2^=Mes, Scheme 1). Path (a) is expected to dominate in the presence of a highly reactive (or concentrated) arene, as e.g. in reactions performed in neat toluene, which would produce the intermediate **10** **tm** (R^1^=Tol, R^2^=Mes). The latter species would undergo one more dearylation, yielding the tolyl‐substituted tropylium [**14** **t**]^+^, and eventually the fully tolylated product **10** **t**. Pathway (b), involving double ionization, may be electrostatically disfavored by the proximity of the cationic units in [**16**]^2+^, which is greater than in other known ditropylium dications.[^45^, ^46^] Path (c) rationalizes the formation of **10** **h** in terms of the known reactivity of tropylium cations as hydride abstractors.^[47]^

**Formation of tropylium cations in superacidic conditions**. We supposed that the PDI tropyliums, postulated above as transarylation intermediates, may form at observable concentrations under strongly acidic conditions. To verify this hypothesis, we carried out experiments in neat triflic acid, which was chosen for its superacidic character[^48^, ^49^] and relative ease of handling. The limited data available on prototropic equilibria of imides in superacids[^50^, ^51^] suggest that PDIs should undergo multiple O‐protonations when dissolved in TfOH. For simplicity, however, the following discussion does not specify the O‐protonation status of the PDI core.

**Figure 4:**
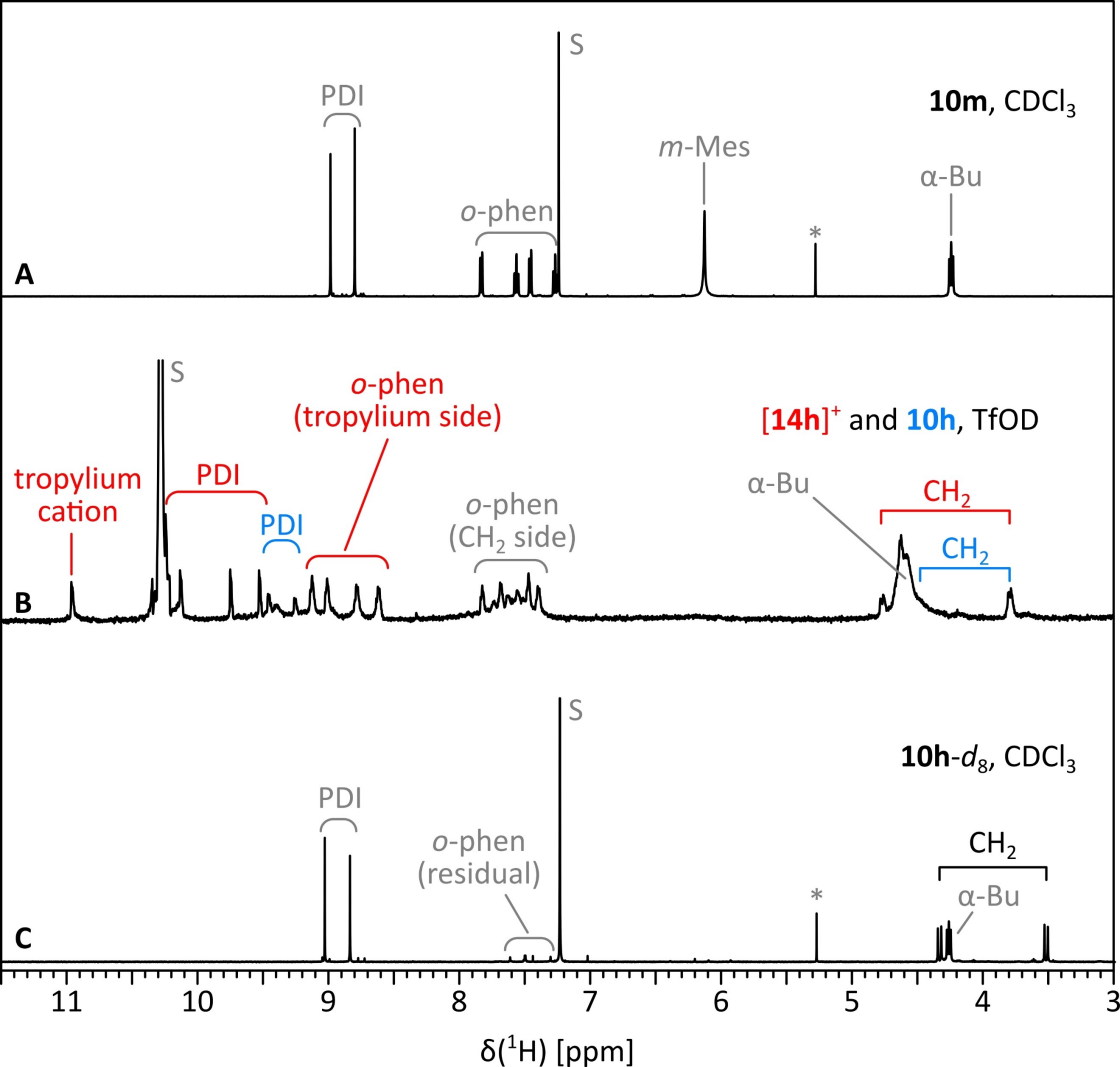
Generation of the tropylium cation [**14** **h**]^+^ monitored using ^1^H NMR spectroscopy. (A) Reference spectrum of **10** **m** (CDCl_3_, 300 K, 500 MHz), (B) Spectrum obtained for **10** **m** (ca. 50 mM) 20 minutes after dissolution in TfOD (300 K, 600 MHz). (C) Spectrum of **10** **h**‐*d*_8_ recovered after quenching the TfOD sample with H_2_O (CDCl_3_, 300 K, 500 MHz). Labels: PDI, perylene CH signals; *o*‐phen, *ortho*‐phenylene signals; CH_2_, sp^3^ bridge of the 7‐membered ring; S, residual solvent signal. O‐protonation/deuteronation is not indicated.

The ^1^H NMR spectrum of **10** **m** recorded ca. 20 min. after dissolution in neat triflic acid‐*d* (TfOD, 300 K, ~50 mM concentration) revealed the presence of the aryl‐free monotropylium cation [**14** **h**]^+^ accompanied by a small amount of the fully hydrogenated species **10** **h** (Figure 4B, Scheme 2C). These two species were identified with the help of 2D correlation spectroscopy. The spectrum of [**14** **h**]^+^ had a lowered symmetry and featured three key resonances identifying the state of seven‐membered rings: a diastereotopic pair of doublets at 4.78 and 3.79 ppm (^2^ *J*_HH_ ≈12 Hz), corresponding to an aryl‐free sp^3^−CH_2_ bridge, and a singlet at 10.97 ppm. The latter signal was assigned to an aromatic tropylium cation on the basis of its uniquely downfield shift, which compared favorably with the corresponding resonance of the dibenzo[*a*,*d*]tropylium cation (10.98 ppm^[52]^). The deshielding seen for *ortho*‐phenylene protons adjacent to the tropylium ring in [**14** **h**]^+^ is similarly consistent with reference data.^[52]^ Upon standing, the concentration of [**14** **h**]^+^ in the TfOD sample decreased and the cation was largely transformed into the dihydro product **10** **h** (Figure S96). Concurrently, both species underwent progressive and highly selective deuteration, which occurred exclusively at the outer *ortho*‐phenylene rings, yielding, respectively, [**14** **h**‐*d*_8_]^+^ and **10** **h**‐*d*_8_. The identity of the latter product was additionally confirmed by isolation from the TfOD solution (Figure 4C) and comparison with the deuterium‐free **10** **h** obtained earlier (cf. Scheme 1).

The rapid formation of [**14** **h**]^+^ in the ^1^H NMR experiment provides an important mechanistic insight into the observed reactivity (Scheme 2C). After the initial dearylation of **10** **m**, the resulting Mes‐substituted tropylium [**14** **m**]^+^ can either (a) undergo one more aryl cleavage, to yield the [**16**]^2+^ dication, or (b) accept a hydride to produce the mono‐Mes intermediate **10** **hm**. The electrophilicity of [**14** **m**]^+^ should be comparable (or lower) than that of [**14** **h**]^+^, implying that the hydride transfer in the [**14** **m**]^+^→**10hm** step should not be faster than in the [**14** **h**]^+^→**10** **h** step, contrary to the experiment. This observation rules out the involvement of **10** **hm**, and provides indirect evidence for the initial formation of the highly electrophilic [**16**]^2+^, which undergoes immediate reduction, to yield [**14** **h**]^+^.

Somewhat surprisingly, the behavior of **9** **tm** in TfOD was different: the ^1^H NMR spectrum of a freshly prepared sample showed that the starting material was intact, with no signs of dearylation or hydrogenation (Figure S107). Over time, however, *ortho*‐phenylene deuteration and gradual decomposition were observed. This result shows that, in contrast to demesitylation of **10** **m**, stoichiometric toluene cleavage from **9** **tm** is not feasible in triflic acid. This difference may be caused by lower thermodynamic stability of the expected tropylium product, [**12** **m**]^+^ (R=Mes, Scheme 2A), or by a lower leaving‐group ability of toluene relative to mesitylene. Nevertheless, equilibrium formation of microscopic amounts of [**12** **m**]^+^ cannot be ruled out, potentially leading to the observed long‐term decomposition.

In the experiments involving TfOD, deuteration of **9** **tm**, **10** **m**, and their derivatives was observed only at the *ortho*‐phenylene rings. The absence of H/D exchange at the perylene core can be rationalized by the electron‐poor character of the PDI substructure. Lack of deuterium incorporation at the 7‐membered rings, notably in the CH_2_ groups of **10** **h**‐*d*_8_, indicates that deuterons (and, by extension, protons) are not the source of hydrogen in the reductive steps. We tested a variety of other solvent/quencher pairs, namely TfOH/H_2_O, TfOH/D_2_O, TfOH/CD_3_OD, and did not observe any CH_2_ deuteration in the resulting **10** **h**. These experiments additionally showed that the tropylium intermediates are more susceptible to reduction than to nucleophile addition, as no hydroxy or alkoxy derivatives were observed. In all of these reactions, the reduction of tropylium cations apparently takes place via hydride transfer involving a deuterium‐free donor. We presume that mesitylene, released in the dearylation steps, may act as a benzylic hydride donor^[53]^ attacking [**14h**]^+^ and [**16**]^2+^ in neat TfOD. The same reactivity is apparently responsible for the formation of the dearylated dianhydride **7** **h** in a reaction involving mesitylene solvent and TfOH catalyst (Scheme 1, reaction **e**). The less electron‐rich toluene seems to favor electrophilic substitution over hydride transfer: a solution of **10** **m** in TfOH produced **10** **t**, when quenched with excess toluene. This result mirrors the synthesis of **7** **t** from **4** **m** (Scheme 1, reaction **c**).

**Scheme 3:**
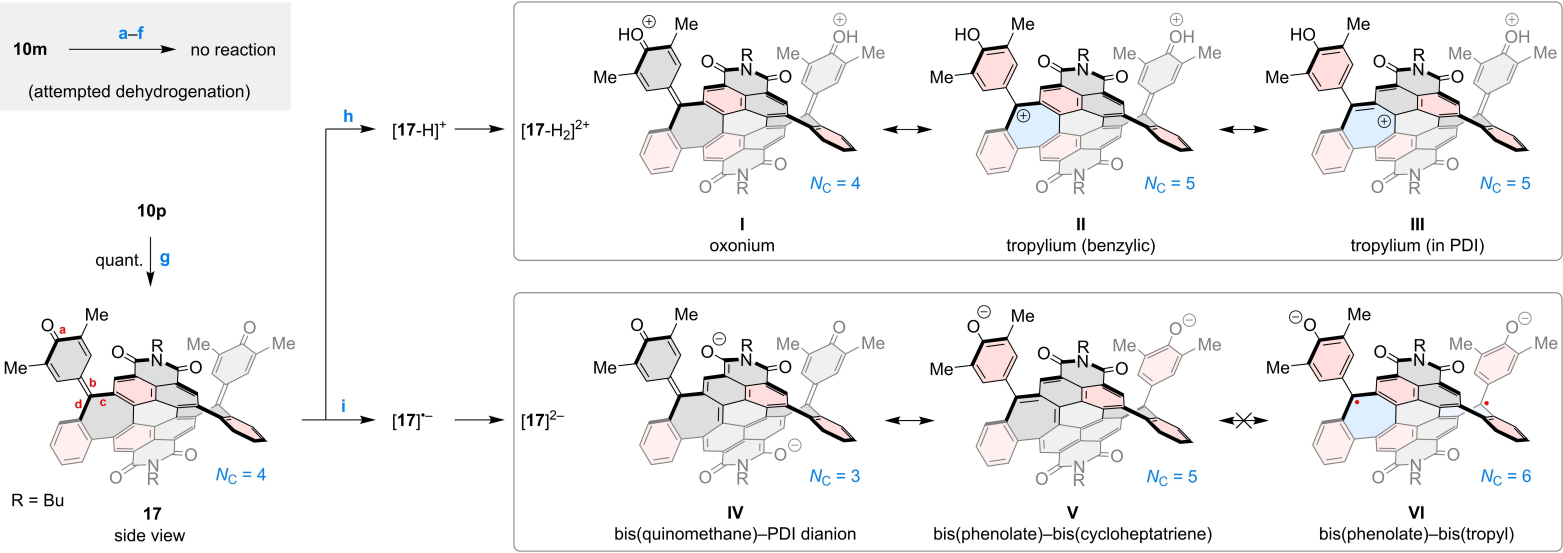
PDI bis(quinomethane) and its reactivity. Reagents and conditions: a) [trityl]BF_4_, MeCN, glove box, 30 min; b) NH_4_NO_3_, DCM, trifluoroacetic anhydride; c) SbCl_5_, DCM, rt; d) FeCl_3_, DCM; e) 1. *t*‐BuOK, THF; 2. I_2_; f) DDQ, DCM, 60 °C, pressure tube; g) DDQ, DCM, rt, 18 h; h) TfOH, 1 : 1 MeCN–DCM, rt; i) CoCp_2_, DCM, rt. *N*_C_ is the number of Clar sextets (shaded in red).

**Figure 5:**
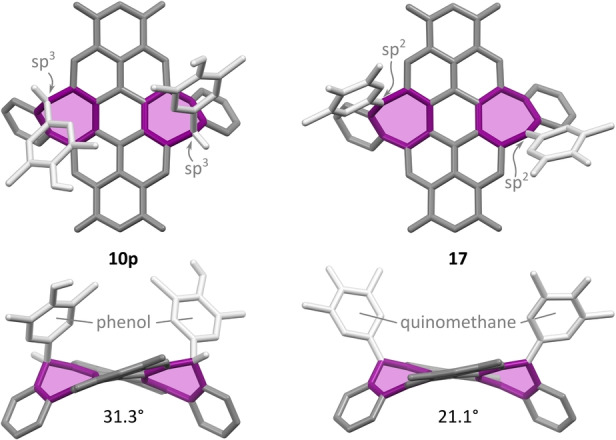
Molecular structures of **10** **p** and **17** determined using single‐crystal X‐ray diffraction analyses. Solvent molecules, N‐butyl substituents, and C(sp^2^)‐bound hydrogens are omitted for clarity. Aromatic backbones are shown in gray, while the C(sp^3^) bridges and the attached substituents are shown in light gray. For each structure, the dihedral angle is given between the mean planes of the C_12_NO_2_ naphthalimide subunits.

**PDI bis(quinomethane)**. To generate PDI tropylium species that would be stable under more conventional conditions and could serve as more accessible models of the transarylation intermediates, we attempted to produce aryl‐substituted cations by direct oxidation of **10** **m**. However, under various conditions (Scheme 3, **a**–**f**), we consistently recovered the unreacted starting material, demonstrating that **10** **m** is highly resistant to dehydrogenation. The origin of this stability can be both thermodynamic, caused by the electron‐deficient character of the PDI core, or kinetic, originating from the unfavorable location of the CH bond relative to the π system.^[13]^ In particular, methods based on initial deprotonation (e.g. route **e**, Scheme 3) are likely unfeasible because of the antiaromatic destabilization of the initial anionic intermediate. In contrast, the phenol‐substituted **10** **p** was cleanly dehydrogenated by DDQ, yielding the bis(quinomethane) **17** (Scheme 3). Here, the dehydrogenation was promoted by the strategic placement of electron‐rich phenol units. The structure of **17**, revealed by X‐ray diffraction, contains two heptafulvenes fused to the PDI core. Its geometry differs noticeably from that of the parent **10** **p** (Figure 5). In contrast to the phenol substituents of **10** **p**, the quinomethane (QM) units[^44^, ^54^, ^55^] in **17** are no longer folded onto the PDI core, which is consequently much less twisted (21.1° vs. 31.3°). Similarly to **10p**, compound **17** is only weakly emissive.

**Figure 6:**
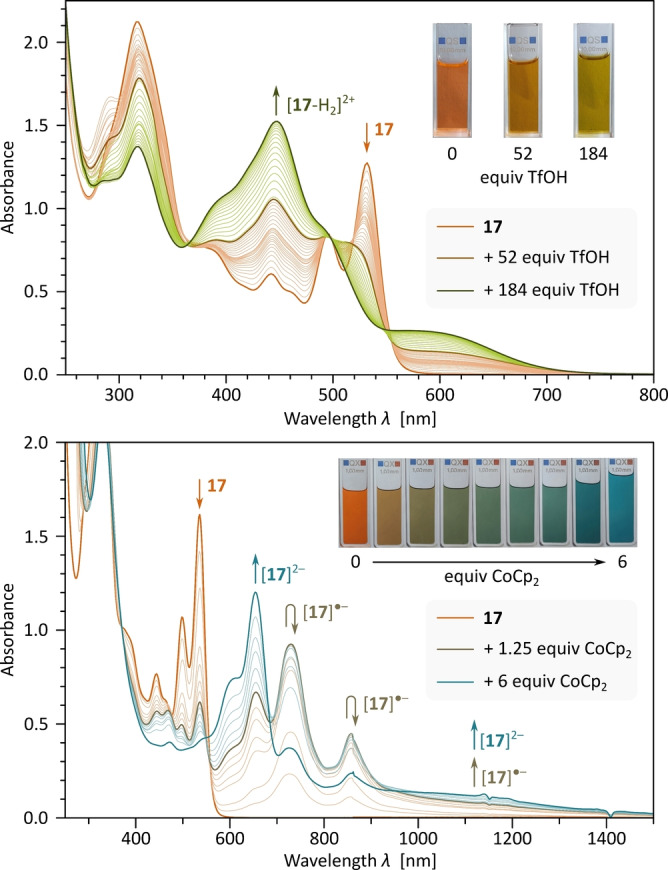
Top: UV/Vis‐NIR spectra of **17** obtained during titration with TfOH (1 : 1 MeCN‐DCM solvent mixture). The intermediate stage corresponding to the maximum concentration of the monocation [**17**‐H]^+^ is indicated with a thick brown line. Bottom: UV/Vis‐NIR spectra of **17** (in deacidified CHCl_3_) obtained during titration with CoCp_2_ in toluene. Spectra corresponding to maximum concentrations of [**17**]⋅^−^ and [**17**]^2−^ were observed for 1.25 and 6 equiv. of CoCp_2_, respectively.

We reasoned that resonance‐stabilized PDI tropylium species could be accessed by addition of electrophiles, protons in particular, to the quinomethane carbonyls of **17**. The latter species was found to be a very weak base and was apparently not protonated by trifluoroacetic acid (Figure S121). However, protonation was achieved with a large excess of TfOH in a 1 : 1 MeCN:DCM mixture (Figure 6, top). The spectral sequence did not contain precise isosbestic points, but it was nevertheless possible to identify two protonation steps by monitoring absorbance changes at 512 nm (Figure S120). The absorption spectra of the corresponding cations, [**17**‐H]^+^ and [**17**‐H_2_]^2+^ (Scheme 3) show similar features, i.e., a strong band at ca. 450 nm, and broad red‐shifted feature in the 560–720 nm range. These spectra differed significantly from that of the neutral **17**, indicating a major change in the chromophore properties caused by protonation.

Electrochemical analyses performed on **17** showed four reversible one‐electron reductions (–1.00, –1.21, –1.82, and –1.91 V vs. Fc^+^/Fc) and one non‐reversible oxidation at ca. 0.93 V (Figure S125). The first two reduction potentials are similar to values reported for fusion‐free PDIs.^[30]^ Chemical reduction of **17** with cobaltocene (CoCp_2_, *E*=–1.3 V^[56]^) produced a two‐stage change of absorption spectra, which was ascribed to sequential formation of radical anion [**17**]⋅^−^ and dianion [**17**]^2−^ (Figure 6, bottom). The reaction could be completely reversed by exposing the reduced solutions to air. The absence of precise isosbestic points and the small difference between the *E*_red1_ and *E*_red2_ potentials imply that the three oxidation levels of **17** coexist in solution. However, specific features of the absorption profile could be confidently assigned to the two anionic states. The spectra of [**17**]⋅^−^ and [**17**]^2−^ bear qualitative resemblance to those of the corresponding PDI anions,[^57^, ^58^] but they also contain broad low‐energy absorption extending in the 900–1500 range. The latter feature may correspond to a charge‐transfer transition involving the PDI core and the QM units. When treated with 6 equiv. of CoCp_2_ in DCM‐*d*_2_ solution, **17** produced a broad ^1^H NMR spectrum at 300 K (Figure S100). The spectrum became sharp upon cooling, showing that the ground state of [**17**]^2−^ is diamagnetic. The room‐temperature broadening most likely results from rapid exchange between [**17**]^2−^ and the paramagnetic [**17**]⋅^−^, rather than from thermal population of the triplet state of the dianion (see below).

The protonated QM units in [**17**‐H]^+^ and [**17**‐H_2_]^2+^ can be described using a range of canonical structures, distributing the positive charge over the π system of the molecule (Scheme 3). Specifically, the charge can be placed not only on the protonated oxygen (**I**) but also delocalized into the seven‐membered rings, either at the benzylic bridge (**II**) or at the peri‐fused positions, including those belonging to the PDI unit (e.g. **III**). Structures such as **II** and **III** have increased Clar sextet counts relative to **I**, and they also contribute to the local aromaticity of the tropylium ring. The involvement of specific substructures in charge delocalization was quantified by analyzing changes of (a) bond lengths in DFT‐optimized geometries of the cations and (b) relative fragment charges Δ*q*_Frag_ ^[59]^ obtained from natural population analysis (NPA, Table 1). [**17**‐H_2_]^2+^ exhibits notable elongation of the **a** and **b** bonds (defined in Scheme 3), consistent with a considerable contribution of non‐quinoidal conjugation. Most of the positive charge is located at the QM fragments (1.59 charge units), however the combined charge on the seven membered rings is substantial (0.30 units), confirming their tropylium‐like character.

**Table 1:** Computational data for relevant states of **17′**.^[a]^

State	**17′** ^[b]^	[**17′**‐H_2_]^2+^	^2^[**17′**]⋅^−^	^1^[**17′**]^2−^	^3^[**17′**]^2− [c]^
a / Å ^[d]^	1.224 (1.250)	1.319	1.229	1.238	1.234, 1.261
b / Å ^[d]^	1.356 (1.367)	1.393	1.361	1.378	1.369, 1.417
c / Å ^[d]^	1.482 (1.488)	1.472	1.478	1.461	1.471, 1.479
d / Å ^[d]^	1.475 (1.478)	1.458	1.476	1.480	1.477, 1.463
Δ*q*_PDI_^[e]^	0.00	0.20	−0.80	−1.43	−0.88
Δ*q*_QM_^[e]^	0.00	1.59	−0.10	−0.38	−0.90
Δ*q*_OP_^[e]^	0.00	0.21	−0.10	−0.19	−0.22
Δ*q*_T_^[e]^	0.00	0.30	−0.14	−0.25	−0.22

[a] CAM−B3LYP/6‐31G(d,p) geometries and densities; **17′** denotes structures with unsubstituted imide nitrogens (R=H, cf. Scheme 3). [b] Corresponding averaged distances of symmetry‐independent bonds in the XRD structure of **17** are given in parantheses. [c] triplet state, bond lengths are given separately for the two non‐equivalent QM units. [d] Bonds a–d are defined in Scheme 3 (red labels). For complete data, see Figure S126. [e] Difference fragment charges defined as Δ*q*_Frag_=*q*_Frag_(**X**) – *q*_Frag_(**17′**), where *q*_Frag_ is a fragment charge obtained as a sum of contributing atomic NPA charges; Frag=PDI, QM, OP, and T corresponds respectively to the PDI, quinomethane, *ortho*‐phenylene, and tropylium (C_7_) units; **X** is the species of interest, and **17′** acts as a reference. Δ*q*_PDI_ + Δ*q*_QM_ + Δ*q*_OP_ sum up to the total system charge.

DFT calculations performed on the dianion [**17**]^2−^ confirmed its singlet ground state, and the thermal inaccessibility of the triplet ^3^[**17**]^2−^ (Δ*E*_ST_ ≈ −14 kcal/mol). The singlet had no open‐shell contributions, in contrast to a structurally similar PDI diradicaloid reported by Würthner et al.^[60]^ The bond‐length pattern obtained for ^1^[**17**]^2−^ suggested that the electronic structure of this species resembles that of fusion‐free PDI dianions, however, the conjugation with the QM moieties is non‐negligible. The latter assumption was confirmed by the NPA charge distribution, which yielded a Δ*q*_QM_ value of –0.38, corresponding to 19 % of the total negative charge. Interestingly, the triplet dianion ^3^[**17**]^2−^ has an asymmetric structure, with a significant portion of negative charge transferred to one of the QM units. Taken together the calculations imply that the conjugation in ^1^[**17**]^2−^ is mostly described by PDI dianionic structures such as **IV** (Scheme 3). The relatively low phenolate contributions (e.g., **V**) and the lack of diradicaloid character (**VI**) can be credited to the highly electron‐deficient nature of the PDI core. Similar conclusions can be drawn for the monoanion [**17**]⋅^−^, for which the involvement of quinomethanes in delocalization is additionally confirmed by the calculated spin density (Figure S127).

## Conclusion

This work presents first examples of perylene diimides featuring five‐ and seven‐membered carbocycles fused at the ortho and bay regions of the perylene core. Our synthetic approach is notable for providing access to three different types of ring fusion from a common, easily accessible precursor. The rigid chiral structures, along with the excellent absorption and emission properties, make the annulated diimides highly promising as chiral chromophores, particularly for applications in circularly polarized luminescence. The PDI cycloheptatrienes can be transformed into π‐conjugated tropylium cations and heptafulvenes, thus opening a new avenue of PDI functionalization. While the tropylium cations reported herein are stable only under strongly acidic conditions, their reactivity toward arenes proves synthetically useful, enabling direct functionalization of the seven‐membered rings. The resistance of cycloheptatriene rings to dehydrogenation can be overcome by incorporating electron‐rich aryl groups, yielding heptafulvenes that can be further transformed into tropylium‐like dications or reduced to mono‐ and dianionic species. The reactivity described here sets the stage for further investigations into penta‐ and heptannulated PDIs, which hold potential as fluorescent emitters, chiral chromophores, and precursors to organic oligoradicaloids. These directions are actively being pursued in our laboratory.

## Supporting Information

The authors have cited additional references within the Supporting Information.[^61^, ^62^, ^63^, ^64^, ^65^, ^66^, ^67^, ^68^, ^69^]

## Conflict of Interests

The authors declare no conflict of interest.

1

## Data Availability

The data that support the findings of this study are available in the supplementary material of this article.

## References

[1] W. Von E. Doering , L. H. Knox , J. Am. Chem. Soc. 1954, 76, 3203–3206.

[2] W. V. Eggers Doering , H. Krauch , Angew. Chem. 1956, 68, 661–667.

[3] G. Merling , Berichte Dtsch. Chem. Ges. 1891, 24, 3108–3126.

[4] K. Komatsu , Chem. Rec. 2015, 15, 160–174. 25346458 10.1002/tcr.201402048

[5] A. E. Asato , X. Y. Li , D. Mead , G. M. L. Patterson , R. S. H. Liu , J. Am. Chem. Soc. 1990, 112, 7398–7399.

[6] K. Asai , A. Fukazawa , S. Yamaguchi , Chem. Eur. J. 2016, 22, 17571–17575. 27684051 10.1002/chem.201604509

[7] K. Asai , A. Fukazawa , S. Yamaguchi , Angew. Chem. Int. Ed. 2017, 56, 6848–6852. 10.1002/anie.201702140 28485833

[8] N. Sprutta , M. Świderska , L. Latos-Grażyński , J. Am. Chem. Soc. 2005, 127, 13108–13109. 16173714 10.1021/ja053723x

[9] N. Sprutta , S. Maćkowiak , M. Kocik , L. Szterenberg , T. Lis , L. Latos-Grażyński , Angew. Chem. Int. Ed. 2009, 48, 3337–3341. 10.1002/anie.200900496 19338003

[10] J. Borstelmann , J. Bergner , F. Rominger , M. Kivala , Angew. Chem. Int. Ed. 2023, 62, e202312740. 10.1002/anie.202312740 37739928

[11] C. Zhu , K. Shoyama , F. Würthner , Angew. Chem. Int. Ed. 2020, 59, 21505–21509. 10.1002/anie.202010077 PMC7756343 32815658

[12] A. Konishi , A. Morinaga , M. Yasuda , Chem. Eur. J. 2018, 24, 8548–8552. 29672951 10.1002/chem.201801915

[13] E. Gońka , P. J. Chmielewski , T. Lis , M. Stępień , J. Am. Chem. Soc. 2014, 136, 16399–16410. 25348930 10.1021/ja508963v

[14] M. Takase , A. Ueno , K. Oki , H. Matsumoto , S. Mori , T. Okujima , H. Uno , Chem. Commun. 2022, 58, 3366–3369. 10.1039/d1cc07152a 35188504

[15] D. J. M. Lyons , R. D. Crocker , M. Blümel , T. V. Nguyen , Angew. Chem. Int. Ed. 2017, 56, 1466–1484. 10.1002/anie.201605979 27506518

[16] W. Xiao , J. Wu , Chin. Chem. Lett. 2023, 34, 107637.

[17] D. J. M. Lyons , R. D. Crocker , T. V. Nguyen , Chem. Eur. J. 2018, 24, 10959–10965. 29774976 10.1002/chem.201801956

[18] R. D. Crocker , D. P. Pace , B. Zhang , D. J. M. Lyons , M. M. Bhadbhade , W. W. H. Wong , B. K. Mai , T. V. Nguyen , J. Am. Chem. Soc. 2021, 143, 20384–20394. 34807589 10.1021/jacs.1c10038

[19] D. Hori , J. H. Yum , H. Sugiyama , S. Park , Bull. Chem. Soc. Jpn. 2021, 94, 1948–1953.

[20] T. Higashino , Y. Fujimori , K. Sugiura , Y. Tsuji , S. Ito , H. Imahori , Angew. Chem. Int. Ed. 2015, 54, 9052–9056. 10.1002/anie.201502951 26080034

[21] K. Horii , R. Kishi , M. Nakano , D. Shiomi , K. Sato , T. Takui , A. Konishi , M. Yasuda , J. Am. Chem. Soc. 2022, 144, 3370–3375. 35188785 10.1021/jacs.2c00476

[22] C. Zhen , S. Lu , M. Lin , J. Wu , I. Chao , C. Lin , Chem. Eur. J. 2021, 27, 16682–16689. 34611945 10.1002/chem.202102781

[23] V. G. Jiménez , P. Mayorga-Burrezo , V. Blanco , V. Lloveras , C. J. Gómez-García , T. Šolomek , J. M. Cuerva , J. Veciana , A. G. Campaña , Chem. Commun. 2020, 56, 12813–12816. 10.1039/d0cc04489j 32966400

[24] A. Shimizu , T. Morikoshi , K. Sugisaki , D. Shiomi , K. Sato , T. Takui , R. Shintani , Angew. Chem. Int. Ed. 2022, 61, e202205729. 10.1002/anie.202205729 35545548

[25] T. Nishiuchi , K. Uchida , T. Kubo , Chem. Commun. 2023, 59, 7379–7382. 10.1039/d3cc02157b 37265413

[26] Y. Hayashi , S. Suzuki , T. Suzuki , Y. Ishigaki , J. Am. Chem. Soc. 2023, 145, 2596–2608. 36606368 10.1021/jacs.2c12574 PMC9896550

[27] L. Moshniaha , M. Żyła-Karwowska , P. J. Chmielewski , T. Lis , J. Cybińska , E. Gońka , J. Oschwald , T. Drewello , S. M. Rivero , J. Casado , M. Stępień , J. Am. Chem. Soc. 2020, 142, 3626–3635. 31997634 10.1021/jacs.9b13942 PMC7467677

[28] Y. Hamamoto , K. Ochiai , Y. Li , E. Tapavicza , S. Ito , Angew. Chem. Int. Ed. 2024, 63, e202319022. 10.1002/anie.202319022 38153357

[29] P. K. Saha , A. Mallick , A. T. Turley , A. N. Bismillah , A. Danos , A. P. Monkman , A.-J. Avestro , D. S. Yufit , P. R. McGonigal , Nat. Chem. 2023, 15, 516–525. 36879076 10.1038/s41557-023-01149-6 PMC10070187

[30] F. Würthner , C. R. Saha-Möller , B. Fimmel , S. Ogi , P. Leowanawat , D. Schmidt , Chem. Rev. 2015, 116, 962–1052. 26270260 10.1021/acs.chemrev.5b00188

[31] A. Nowak-Król , K. Shoyama , M. Stolte , F. Würthner , Chem. Commun. 2018, 54, 13763–13772. 10.1039/c8cc07640e 30465555

[32] R. Mishra , P. Panini , J. Sankar , Org. Lett. 2014, 16, 3994–3997. 25033413 10.1021/ol501822c

[33] S. Hayakawa , A. Kawasaki , Y. Hong , D. Uraguchi , T. Ooi , D. Kim , T. Akutagawa , N. Fukui , H. Shinokubo , J. Am. Chem. Soc. 2019, 141, 19807–19816. 31746597 10.1021/jacs.9b09556

[34] M. Odajima , K. Tajima , N. Fukui , H. Shinokubo , Angew. Chem. Int. Ed. 2021, 60, 15838–15843. 10.1002/anie.202104882 33928728

[35] Y. Tanaka , K. Matsuo , H. Yamada , N. Fukui , H. Shinokubo , Eur. J. Org. Chem. 2022, 2022, e202200770.

[36] K. Fujimoto , S. Izawa , A. Takahashi , T. Inuzuka , K. Sanada , M. Sakamoto , Y. Nakayama , M. Hiramoto , M. Takahashi , Chem. Asian J. 2021, 16, 690–695. 33491273 10.1002/asia.202100066

[37] F. Brust , O. Nagler , K. Shoyama , M. Stolte , F. Würthner , Adv. Opt. Mater. 2023, 11, 2202676.

[38] S. Sengupta , R. K. Dubey , R. W. M. Hoek , S. P. P. van Eeden , D. D. Gunbaş , F. C. Grozema , E. J. R. Sudhölter , W. F. Jager , J. Org. Chem. 2014, 79, 6655–6662. 24984205 10.1021/jo501180a

[39] A. H. G. David , M. Roger , O. Alévêque , H. Melnychenko , L. Le Bras , M. Allain , A. Gapin , D. Canevet , O. Ségut , E. Levillain , A. Goujon , Angew. Chem. Int. Ed. n.d., n/a, e202413616. 10.1002/anie.202413616 PMC11701345 39163166

[40] H. Langhals , P. von Unold , Angew. Chem. Int. Ed. Engl. 1995, 34, 2234–2236.

[41] “2378449 (**8** **m**), 2377911 (**9** **tm**), 2377900 (**10** **m**), 2377912 (**10** **p**), 2381349 (**10** **h**), 2377914 (**17**) contain the supplementary crystallographic data for this paper. These data are provided free of charge by the joint Cambridge Crystallographic Data Centre and Fachinformationszentrum Karlsruhe Access Structures service.,”.

[42] A. Borissov , P. J. Chmielewski , C. J. Gómez García , T. Lis , M. Stępień , Angew. Chem. Int. Ed. 2023, e202309238. 10.1002/anie.202309238 37452009

[43] J. J. Looker , J. Org. Chem. 1965, 30, 4180–4183.

[44] J. J. Looker , J. Org. Chem. 1967, 32, 2941–2942.

[45] A. Kagayama , K. Komatsu , T. Nishinaga , K. Takeuchi , C. Kabuto , J. Org. Chem. 1994, 59, 4999–5004.

[46] K. Komatsu , T. Nishinaga , N. Maekawa , A. Kagayama , K. Takeuchi , J. Org. Chem. 1994, 59, 7316–7321.

[47] J. L. Miller , J.-M. I. A. Lawrence , F. O. R. del Rey , P. E. Floreancig , Chem. Soc. Rev. 2022, 51, 5660–5690. 35712818 10.1039/d1cs01169c

[48] R. D. Howells , J. D. Mc Cown , Chem. Rev. 1977, 77, 69–92.

[49] G. A. Olah, G. K. Surya Prakash, in *Encycl. Phys. Sci. Technol. Third Ed*. (Ed.: R. A. Meyers), Academic Press, New York, **2003**, pp. 175–188.

[50] G. A. Olah , R. H. Schlosberg , J. Am. Chem. Soc. 1968, 90, 6464–6467.

[51] K. Yu. Koltunov , G. K. S. Prakash , G. Rasul , G. A. Olah , Eur. J. Org. Chem. 2006, 2006, 4861–4866.

[52] G. A. Olah , G. Liang , J. Org. Chem. 1975, 40, 2108–2116.

[53] N. C. Deno , H. J. Peterson , G. S. Saines , Chem. Rev. 1960, 60, 7–14.

[54] T. Nozoe , K. Takahashi , Bull. Chem. Soc. Jpn. 1967, 40, 1473–1479.

[55] B. Föhlisch , P. Bürgle , D. Krockenberger , Chem. Ber. 1968, 101, 2717–2730.

[56] N. G. Connelly , W. E. Geiger , Chem. Rev. 1996, 96, 877–910. 11848774 10.1021/cr940053x

[57] R. Renner , M. Stolte , J. Heitmüller , T. Brixner , C. Lambert , F. Würthner , Mater. Horiz. 2022, 9, 350–359. 34816838 10.1039/d1mh01019k

[58] H. Li , O. S. Wenger , Angew. Chem. Int. Ed. 2022, 61, e202110491. 10.1002/anie.202110491 PMC9299816 34787359

[59] H. Zhylitskaya , J. Cybińska , P. Chmielewski , T. Lis , M. Stępień , J. Am. Chem. Soc. 2016, 138, 11390–11398. 27533895 10.1021/jacs.6b07826

[60] D. Schmidt , M. Son , J. M. Lim , M.-J. Lin , I. Krummenacher , H. Braunschweig , D. Kim , F. Würthner , Angew. Chem. Int. Ed. 2015, 54, 13980–13984. 10.1002/anie.201507039 26350026

[61] T. Chand , L. Khamari , D. Chopra , S. Mukherjee , M. Kapur , Chem. Eur. J. 2022, 28, e202200723. 35561125 10.1002/chem.202200723

[62] G. R. Fulmer , A. J. M. Miller , N. H. Sherden , H. E. Gottlieb , A. Nudelman , B. M. Stoltz , J. E. Bercaw , K. I. Goldberg , Organometallics 2010, 29, 2176–2179.

[63] M. J. Frisch, G. W. Trucks, H. B. Schlegel, G. E. Scuseria, M. A. Robb, J. R. Cheeseman, G. Scalmani, V. Barone, G. A. Petersson, H. Nakatsuji, X. Li, M. Caricato, A. F. Izmaylov, G. Zheng, J. L. Sonnenberg, M. Hada, M. Ehara, K. Toyota, R. Fukuda, J. Hasegawa, M. Ishida, T. Nakajima, Y. Honda, O. Kitao, H. Nakai, T. Vreven, J. A. Montgomery, Jr., J. E. Peralta, F. Ogliaro, M. Bearpark, J. J. Heyd, E. Brothers, K. N. Kudin, V. N. Staroverov, R. Kobayashi, J. Normand, K. Raghavachari, A. Rendell, J. C. Burant, S. S. Iyengar, J. Tomasi, M. Cossi, J. M. Millam, M. Klene, C. Adamo, R. Gomperts, R. E. Stratmann, O. Yazyev, A. J. Austin, R. Cammi, C. Pomelli, J. W. Ochterski, R. L. Martin, K. Morokuma, V. G. Zakrzewski, G. A. Voth, P. Salvador, J. J. Dannenberg, S. Dapprich, A. D. Daniels, O. Farkas, J. B. Foresman, D. J. Fox, **2016**.

[64] A. D. Becke , Phys. Rev. A 1988, 38, 3098–3100. 10.1103/physreva.38.3098 9900728

[65] A. D. Becke , J. Chem. Phys. 1993, 98, 5648–5652.

[66] C. Lee , W. Yang , R. G. Parr , Phys. Rev. B 1988, 37, 785–789. 10.1103/physrevb.37.785 9944570

[67] T. Yanai , D. P. Tew , N. C. Handy , Chem. Phys. Lett. 2004, 393, 51–57.

[68] S. Grimme , S. Ehrlich , L. Goerigk , J. Comput. Chem. 2011, 32, 1456–1465. 21370243 10.1002/jcc.21759

[69] A. E. Reed , R. B. Weinstock , F. Weinhold , J. Chem. Phys. 1985, 83, 735–746.

